# Supplementary material for: Maximizing binary interactome mapping with a minimal number of assays
Source: Nat Commun. 2019 Aug 29;10:3907. doi: 10.1038/s41467-019-11809-2 (PMC6715725; doi:10.1038/s41467-019-11809-2)
Supplement: Supplementary file 1 — Description of Additional Supplementary Files [file 41467_2019_11809_MOESM1_ESM.docx]

**Title:** Supplementary Data 1
**Description:** Classification of PPI detection methods as binary or non-binary and pairs discarded from hsPRS-v1 and hsRRS-v1. Classification of PPI detection methods is presented in sheet 1 and list of discarded v1 pairs (with reason for removal) in sheet 2.

**Title:** Supplementary Data 2
**Description:** Literature information supporting hsPRS-v2 pairs. PubMed IDs are presented for each protein pair.

**Title:** Source Data
**Description:** hsPRS-v2 and hsRRS-v2 data for all assay versions tested in the present study. All data for 26 assay versions averaged from three replicates are presented in sheet one. All N2H raw data including three replicates of tests and empty controls are presented in the second sheet.
